# Significance of CD8+T cells related gene *ITGB2* in prognosis and tumor microenvironment of small cell lung cancer

**DOI:** 10.1097/MD.0000000000041461

**Published:** 2025-02-14

**Authors:** Nana Wang, Wen Tian, Jinhui Zhao, Wenzhong Wang, Fu Mi

**Affiliations:** a Department of General Internal Medicine, Tianjin Hospital, Tianjin, China; b The Second Department of Medical Oncology, Cangzhou Central Hospital, Cangzhou, China; c Department of Comprehensive Treatment, Hejian Branch of Cangzhou Central Hospital, Cangzhou, China; d Department of Medical Oncology, Huanxing Cancer Hospital of Chaoyang District, Beijing, China; e The Second Department of Medical Oncology, Tianjin Medical University, Tianjin, China.

**Keywords:** CD8 + T cells, immune checkpoints, *ITGB2*, prognosis, small cell lung cancer

## Abstract

The CD8 + T cells could enhance response of antitumor immune in cancers. Therefore, we aimed to analyze the CD8 + T cells related genes in small cell lung cancer (SCLC) patients. The data of SCLC samples were collected from the Gene Expression Omnibus database. The hub genes were screened by weighted gene co-expression network analysis, protein–protein interaction network and survival analyses. Moreover, the relative proportions of the 22 immune cells in the samples were calculated using CIBERSORT software. The relationship between target gene and immunotherapy was analyzed in IMvigor210 cohort. We identified 10 genes (*PTPRC*, *RPS27A*, *UBA52*, *CD8A*, *ITGB2*, *GNB2L1*, *TYROBP*, *CD86*, *TLR4*, and *FCGR3A*) that were correlated with CD8 + T cells in SCLC. Among them, *ITGB2* was positively correlated with CD8 + T cells in SCLC. *ITGB2* was down-regulated in SCLC. SCLC patients with low *ITGB2* expression exhibited a poor prognosis, and *ITGB2* was an independent prognostic factor for survival rate of SCLC patients. The differentially expressed genes between *ITGB2*^high^ and *ITGB2*^low^ groups were significantly enriched in 143 signaling pathways. We also discovered that the ImmuneScore, StromalScore, and the expression of immune checkpoints (PD-1 (PDCD1), CTLA4, PDL-1 (CD274), TIGIT, IFNG, GZMA, TBX2, and IDO1) were significantly increased in *ITGB2*^high^ group. Moreover, SCLC patients with high *ITGB2* expression had lower tumor immune dysfunction and exclusion scores, and the proportion of urothelial cancer patients with complete response/partial response was observably decreased in *ITGB2*^high^ group. Finally, we found that *ITGB2* was correlated with IC50 of cancer drugs. In conclusion, SCLC patients with low *ITGB2* expression exhibited worse prognosis. *ITGB2* might be correlated with immunotherapy response of SCLC patients.

## 1. Introduction

Epidemiological investigation has revealed that lung cancer is the tumor with the highest incidence and mortality in China.^[1]^ As one of the important pathological types of lung cancer, small cell lung cancer (SCLC) is characterized by high invasiveness, rapid progression, and poor prognosis, with a median overall survival of just 9 to 10 months and a 5-year survival rate of <5%.^[2,3]^ Comparing to the non-small cell lung cancer (NSCLC), fewer drugs have been approved for the treatment of SCLC.^[4,5]^ Currently, no effective molecular targeted agents have been found to significantly prolong the survival rate of SCLC patient.^[6]^ In addition, the key biomarkers and specific targets for SCLC prognosis remain unclear because of the complexity of its biological properties. Therefore, it is critical to investigate the molecular mechanisms of SCLC, development, and uncover possible biomarkers for targeted treatment in order to enhance patients’ clinical efficacy.

The use of immune checkpoint inhibitors has revolutionized the SCLC treatment landscape. For example, the combination of anti-PDL-1 antibody with platinum-based doublet chemotherapy has emerged as first-line therapy for a wide range of SCLC.^[7]^ Unfortunately, only a subset of SCLC patients seemed to obtain benefit.^[8]^ One study evaluated the efficacy and safety of ipilimumab in combination with etoposide and platinum in extensive stage SCLC patients, but the overall survival of patients was not significantly prolonged following treatment.^[9]^ It has been showed that the tumor microenvironment (TME) may be associated with the efficacy of immunotherapy for SCLC. The TME is a complex system consisting of multiple distinct cell populations and noncellular materials. They include many components, such as immune cells, fibroblasts, stromal, and a variety of metabolites and cytokines.^[10]^ Infiltrating immune cells are critical for antitumor efficacy. For example, the depletion of natural killer cells is found to significantly enhance the metastatic spread of SCLC tumor cells in vivo. Conversely, the administration of anti-PD-L1 treatment in SCLC mice resulted in substantial activation and increased infiltration of natural killer cells within the TME, consequently playing a crucial role in suppressing tumor metastasis.^[11]^

Tumor-infiltrating lymphocytes (TILs) are one of the most important components of tumor-infiltrating immune cells in the TME. It has been reported that high number of TILs has been associated to a good prognosis^[12,13]^ and a high potential of responding to immune checkpoint inhibitors in certain cancers.^[14,15]^ CD8 + cytotoxic T lymphocytes are the most important TILs subset because these cells can directly kill cancer cells.^[16]^ Moreover, high densities of CD8 + T cell have been linked to a better prognosis in a variety of tumor types.^[17,18]^ In SCLC, the depletion of CD8 + T cells reverses the antitumor efficacy of PARP and CHK1 inhibitors,^[19]^ whereas increasing the number and function of CD8 + T cells enhances antitumor immune responses.^[20]^ Currently, several immunotherapeutic targets have been identified in CD8 + T cells,^[21]^ suggesting that CD8 + T cells may be deemed as a potential avenue for antitumor. In addition, it has been demonstrated that markers associated with CD8 + T cells exhibit great potential for advancing immunotherapy and predicting prognosis in multiple cancers.^[22,23]^ Whereas few reports have revealed the effect of CD8 + T cells in SCLC to our knowledge. Hence, the identification of CD8 + T cells related markers in SCLC will facilitate the exploration of immune infiltration mechanisms and enrich immunotherapy for SCLC.

## 2. Materials and methods

### 2.1. Data source

Three datasets (GSE43346, GSE30219, and GSE60052) were downloaded from the Gene Expression Omnibus (https://www.ncbi.nlm.nih.gov/geo/) database. GSE43346 included 23 SCLC and 42 normal samples, GSE30219 contained 21 SCLC and 14 normal samples, GSE60052 had 79 SCLC (49 samples with complete survival information) and 7 normal samples. Ethical approval was not required for this study because all analyses were performed using publicly available data.

### 2.2. Weighted gene co-expression network analysis (WGCNA)

WGCNA was performed using the “WGCNA” function package^[24]^ in R language (version 4.2.1, the same below) according to the expression values of genes, and the top 25% of the values of gene expression were screened for WGCNA by analysis of variance. The Pearson correlation coefficients were calculated for each gene, and appropriate soft thresholds were selected β. One-step was applied to build gene network, and the adjacency matrix was transformed into topological overlap matrix, and hierarchical clustering was applied to produce hierarchical clustering tree. The significances of genes and model were calculated to measure the significance between gene and clinical information, and the significant association between module and traits was analyzed to obtain gene module.

### 2.3. Functional enrichment analysis

The differentially expressed genes were performed to enrichment analysis (including Gene Ontology (GO) term and Kyoto Encyclopedia of genes and Genomes (KEGG) pathway) using “Clusterprofiler”^[25]^ function package in the R language. The significantly enriched pathways were screened according to *P* < .05.

### 2.4. Protein–protein interaction (PPI) networks analysis

STRING^[26]^ (https://string-db.org/,version 11.0) was applied to analyze the functional associations of proteins and protein interactions. The PPI was visualized using Cytoscape^[27]^ (version 3.7.2), and the algorithm based on maximum neighbor component was employed to further screen the hub genes in the PPI network with the cytohubba plugin in Cytoscape software.

### 2.5. Immune cell infiltration

SCLC samples in the Gene Expression Omnibus database were used to performed the immune cell infiltration analysis. The relative proportions of the 22 immune cells in the samples were calculated using CIBERSORT software.^[28]^ CIBERSORT software characterizes the composition of immune infiltrating cells based on gene expression matrices using a deconvolution algorithm using a preset set of 547 barcode genes. In addition, the TIMER2.0 database^[29]^ was used to calculate the amounts of each type of immune infiltrating cells in the samples. The TIMER2.0 database integrates xCELL (https://comphealth.ucsf.edu/app/xcell) and QUANTISEQ (https://icbi.i-med.ac.at/software/quantiseq/doc/index.html) and other algorithms, can yield richer results. The ImmuneScore and StromalScore were calculated using “estimate” function package, and response of patients to immunotherapy was assessed with the tumor immune dysfunction and exclusion (TIDE) score (http://tide.dfci.harvard.edu/).

### 2.6. Survival analysis

The overall survival of different groups was evaluated by survival and survminer packages (https://CRAN.R-project.org/package=survival) in the R language, based on the Kaplan–Meier method. The log-rank test was used to test the significance of differences in survival between different groups.

### 2.7. Drug sensitivity analysis

CalcPhenotype was a function in oncoPredict that was an R package specifically designed for drug response prediction. Calcphenotype used large-scale gene expression and drug screening data to build a ridge regression model, which was then applied to new gene expression datasets, enabling prediction of clinical chemotherapy response. Moreover, Calcphenotype integrates models derived from GDSC or CTRP data as training data sets, and users can directly input gene expression data for the prediction of drug sensitivity. The IC50 of 198 drugs were calculated using the oncoPredict^[30]^ function package, and analyzed the correlation between target gene and IC50 of drugs.

### 2.8. Prediction of immunotherapy outcome

IMvigor210 was an open multicenter, single-arm phase 2 clinical study, which was a cohort study of atezolizumab in patients with locally advanced or metastatic urothelial carcinoma. IMvigor210 included 310 metastatic urothelial cancer patients, the PD-1 expressions in tumor-infiltrating immune cells of each patient were detected by SP142 immunohistochemistry. The relationship between target gene and immunotherapy was analyzed using IMvigor210 cohort.

### 2.9. Statistical analysis

The Wilcoxon rank sum test (two-sided) was employed to analyze the differences in infiltration of immune cells between different groups, no multiple comparisons were done here. Spearman correlation analysis was performed using the R language “cor” function. *P* < .05 was considered statistically significant. R software was used for all statistical analyses.

## 3. Results

### 3.1. Identification of central modules associated with CD8 + T cells in SCLC samples

**Figure 1.**
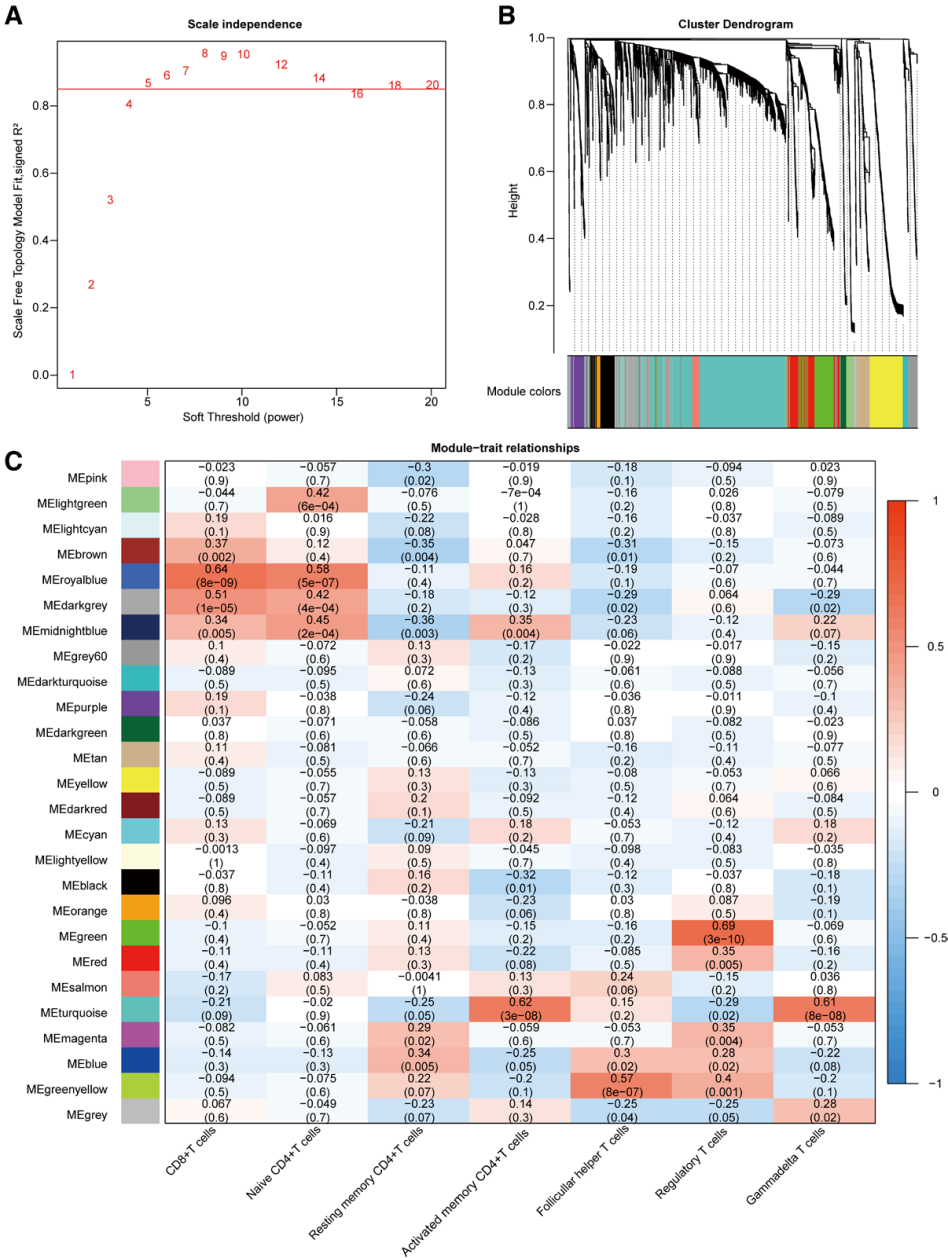
The results of WGCNA and enrichment analysis. (A) Determine the soft threshold β. (B) The result of gene module clustering, the top half is a hierarchical clustering tree of genes, the bottom half is the gene module, module colors represent the color of each module, gray is the collection of genes that cannot be aggregated into other modules. (C) The correlation of modules and traits. WGCNA = weighted gene co-expression network analysis.

In the GSE43346 dataset, we performed the WGCNA and selected the soft threshold β = 5 (Fig. 1A). We constructed a gene network and obtained 26 gene modules (Fig. 1B). We also calculated the relative proportions of the 22 immune cells in the samples and selected the fractions of the 7 subtypes of T cells in each sample as trait data for the WGCNA. Next, we calculated the correlation between the gene modules and the infiltration levels of 7 T cell subtypes: CD8 + T cells, naïve CD4 + T cells, resting memory CD4 + T cells, activated memory CD4 + T cells, follicular helper T cells, regulatory T cells, gammadelta T cells (Fig. 1C). We found that Royalblue, DarkGray, Brown and Midlightblue gene modules were significantly positively correlated with CD8 + T cells (Fig. 1C). Therefore, these 4 gene modules were selected as key modules, containing a total of 609 genes.

**Figure 2.**
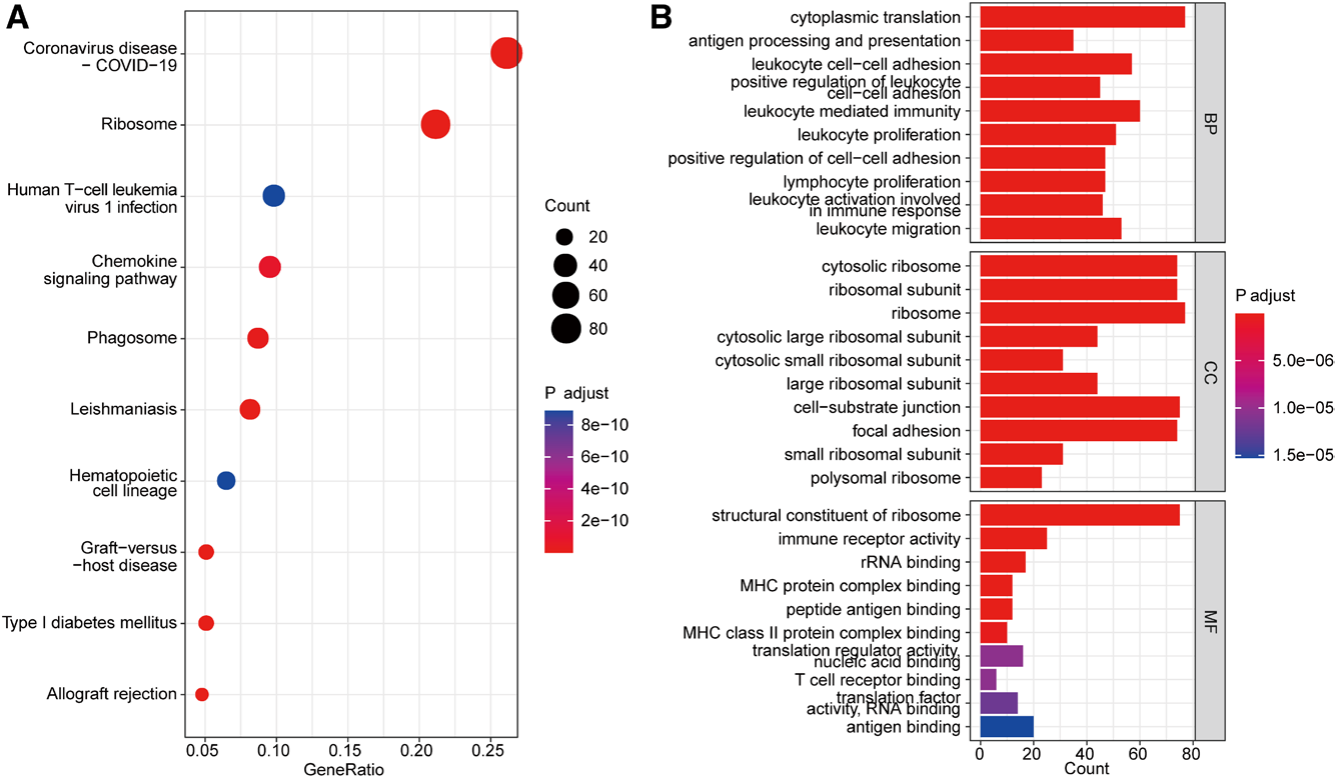
Functional enrichment analysis results of marker genes. (A) The top 10 significantly enriched KEGG pathways. (B) The top 10 significantly enriched in BP, CC, and MF terms. BP = biological process, CC = cellular component, KEGG = Kyoto Encyclopedia of genes and Genomes, MF = molecular function.

Next, we performed KEGG and GO enrichment analysis to explore the functional enrichment of these 609 genes in SCLC. We found that these 609 genes were remarkably enriched in 66 KEGG pathways, such as human T cell leukemia virus 1 infection, chemokine signaling pathway, and leishmaniasis (Table S1, Supplemental Digital Content, http://links.lww.com/MD/O353, Fig. 2A). The GO analysis showed that these 609 genes were significantly enriched in 840 biological process (BP) terms, 68 molecular function (MF), and 102 cellular component (CC) terms (Table S1, Supplemental Digital Content, http://links.lww.com/MD/O353), including leukocyte cell–cell adhesion positive regulation of leukocyte cell–cell adhesion, immune receptor activity, and T cell receptor binding (Fig. 2B). The top 10 significantly enriched pathways in KEGG and GO (BP, CC, and MF) were displayed in Figure 2A and B, respectively.

### 3.2. Identification of hub genes in central modules in SCLC samples

**Figure 3.**
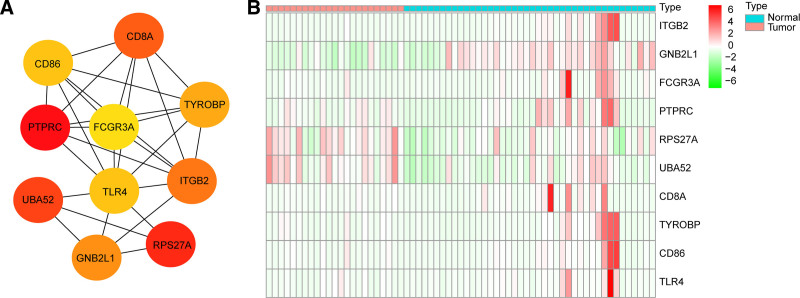
Identification of CD8 + T cell-related hub genes. (A) Protein network interaction map of top10 genes. (B) The expression of top 10 genes.

Based on these 609 genes, we constructed a PPI network to screen key genes, and the minimum required interaction score > 0.4 as the threshold to select the interaction pairs. A total of 554 nodes and 9978 edges were displayed in PPI network (Figure S1, Supplemental Digital Content, http://links.lww.com/MD/O352), and the top 10 genes (*PTPRC*, *RPS27A*, *UBA52*, *CD8A*, *ITGB2*, *GNB2L1*, *TYROBP*, *CD86*, *TLR4*, and *FCGR3A*) were selected as hub CD8 + T cell-related genes in SCLC (Fig. 3A, Table S2, Supplemental Digital Content, http://links.lww.com/MD/O353). Among these 10 genes, *ITGB2*, *GNB2L1*, and *FCGR3A* expression was decreased in SCLC samples in the GSE43346 dataset (Fig. 3B).

### 3.3. Correlation between hub genes and CD8 + T cells, and establishment of the PPI network for transcription factor genes

**Figure 4.**
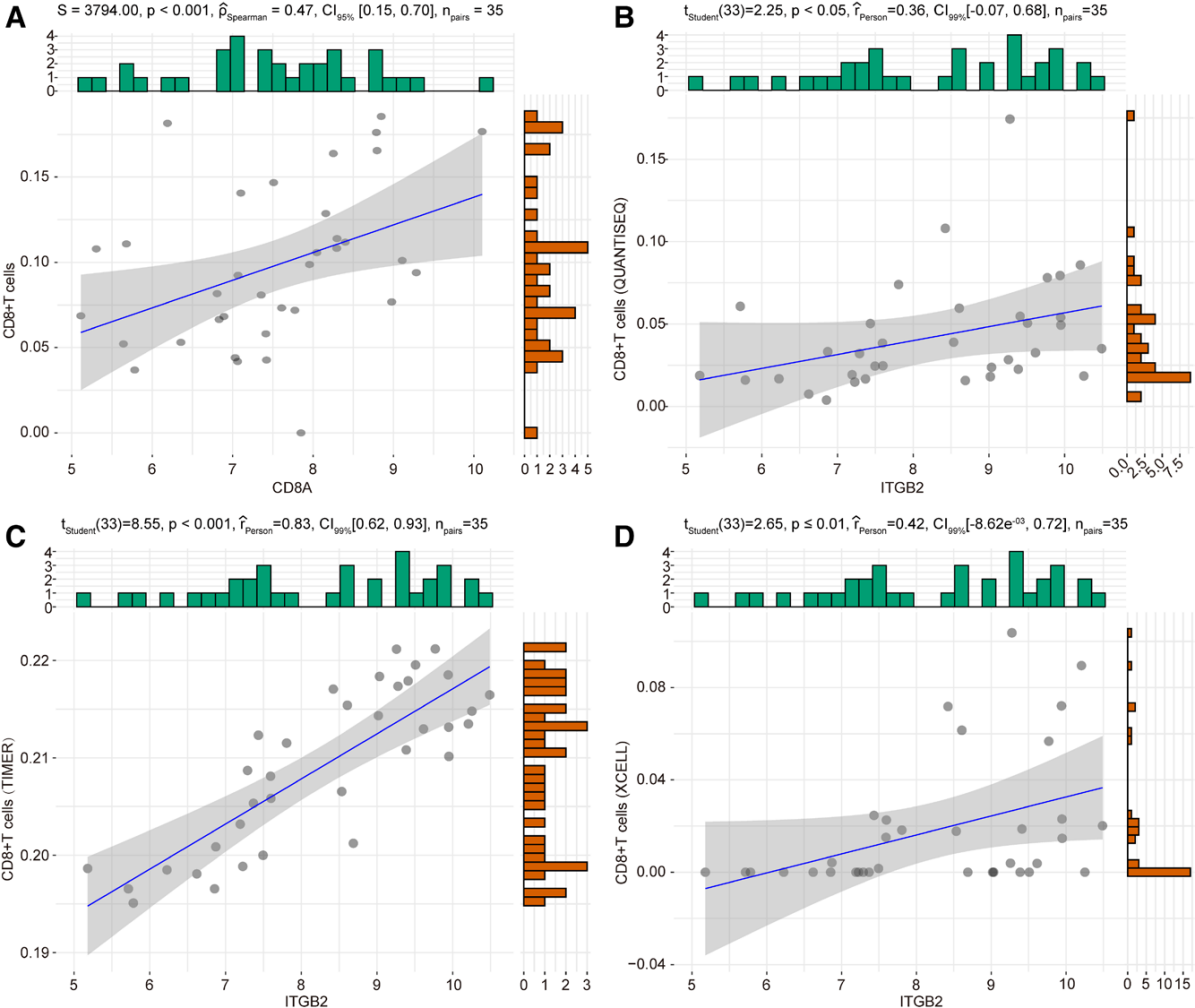
Verification results of hub gene. (A) The correlation of CD8 + T cells with *CD8A*. (B–D) The correlation of CD8 + T cells with *ITGB2*.

**Figure 5.**
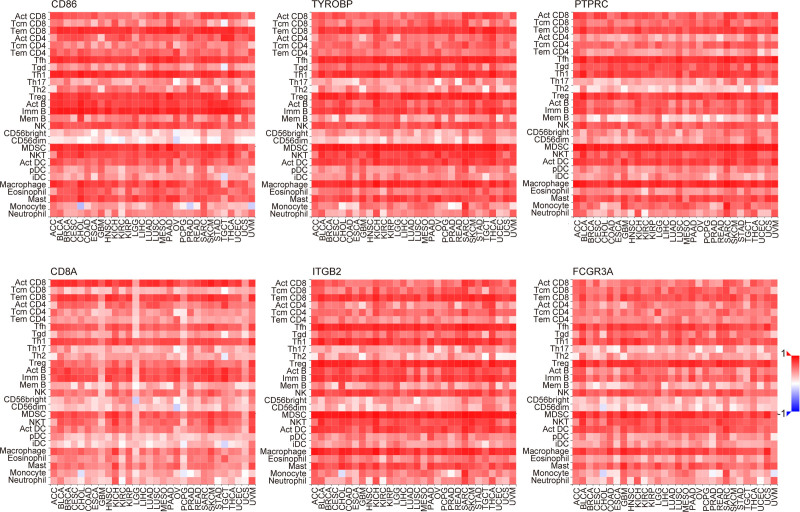
The correlation of 6 hub genes (*PTPRC*, *CD86*, *TYROBP*, *CD8A*, *ITGB2*, and *FCGR3A*) with tumor-infiltrating lymphocyte in pan-cancer.

Next, to explore the association of 10 genes with CD8 + T cells, we analyzed the spearman correlation between these 10 genes and CD8 + T cells. As shown in Figure 4A, the spearman correlation analysis showed that CD8A had positive correlation with CD8 + T cell among these 10 hub genes (*P* < .001, *R* = 0.47). In the GSE30219 dataset, *ITGB2* was significantly positively correlated with CD8 + T cells as estimated by the QUANTISEQ, TIMER, and Xcell algorithms (Fig. 4B–D). Moreover, the univariate cox regression analysis showed that the CD8 + T cells were correlated with prognosis of SCLC patients in TIMER database (Figure S2A, Supplemental Digital Content, http://links.lww.com/MD/O352). In pan-cancer, *PTPRC*, *CD86*, *TYROBP*, *CD8A*, *ITGB2*, *FCGR3A* were also highly correlated with active CD8 + T cells (Act CD8) (Fig. 5, Figure S3A–S3D, Supplemental Digital Content, http://links.lww.com/MD/O352).

Subsequently, the module genes were enriched to transcription factors using Gene Functional Classification function in the DAVID (https://david.ncifcrf.gov/) database. A total of 609 genes in central module were enriched to 16 transcription factors (Table S3, Supplemental Digital Content, http://links.lww.com/MD/O353). Next, a PPI network was built using 16 transcription factor genes, and this PPI network contained 9 nodes and 22 edges (Figure S2B, Supplemental Digital Content, http://links.lww.com/MD/O352).

### 3.4. ITGB2 was an independent prognostic factor for survival of SCLC patients

**Figure 6.**
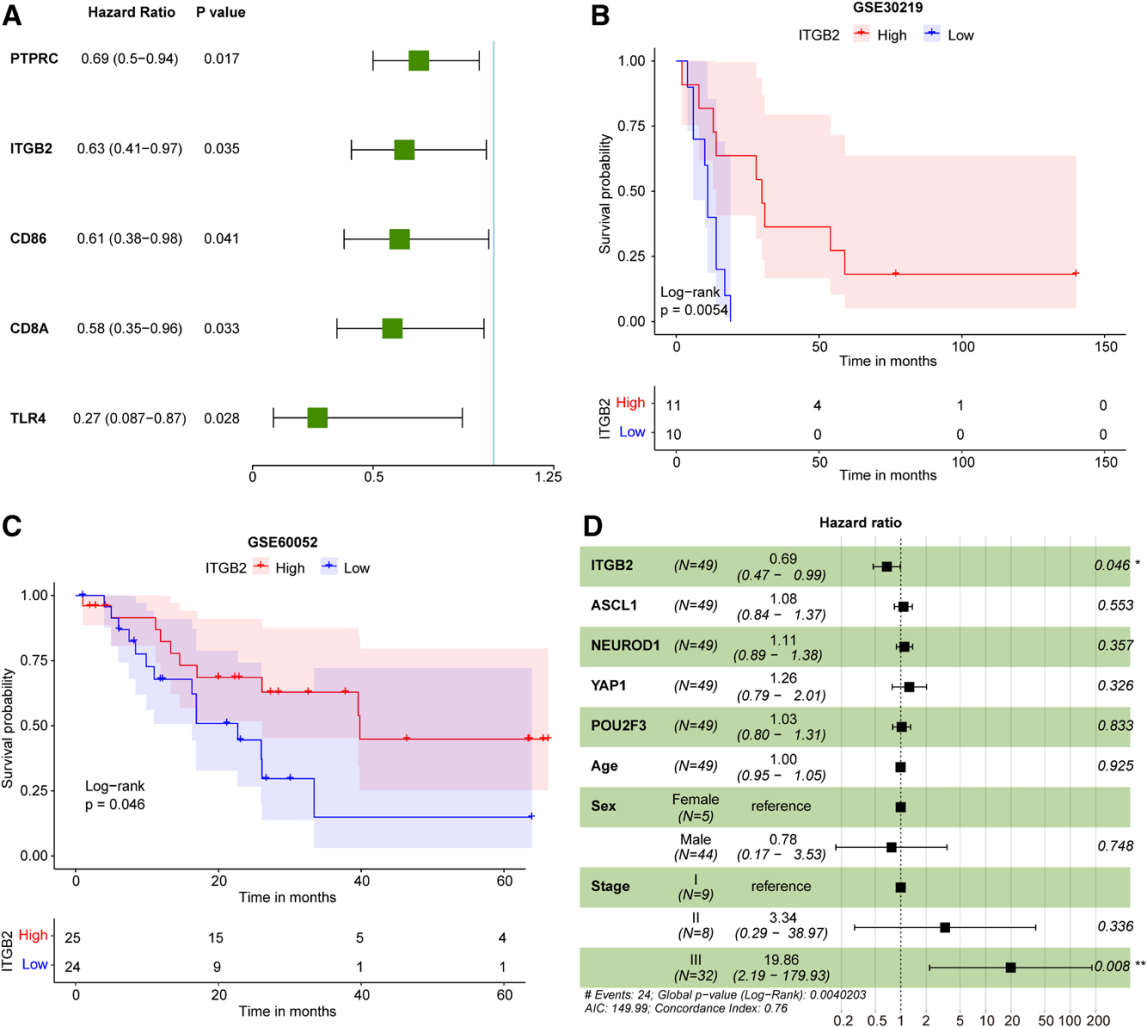
The result of survival analysis. (A) Forest map of univariate cox analysis. (B and C) Kaplan–Meier survival curves of *ITGB2*^high^ and *ITGB2*^low^ groups in GSE30219 and GSE60052 datasets. (D) The multivariate Cox regression analysis included *ITGB2*, age, sex, and stage in GSE60052 dataset.

**Figure 7.**
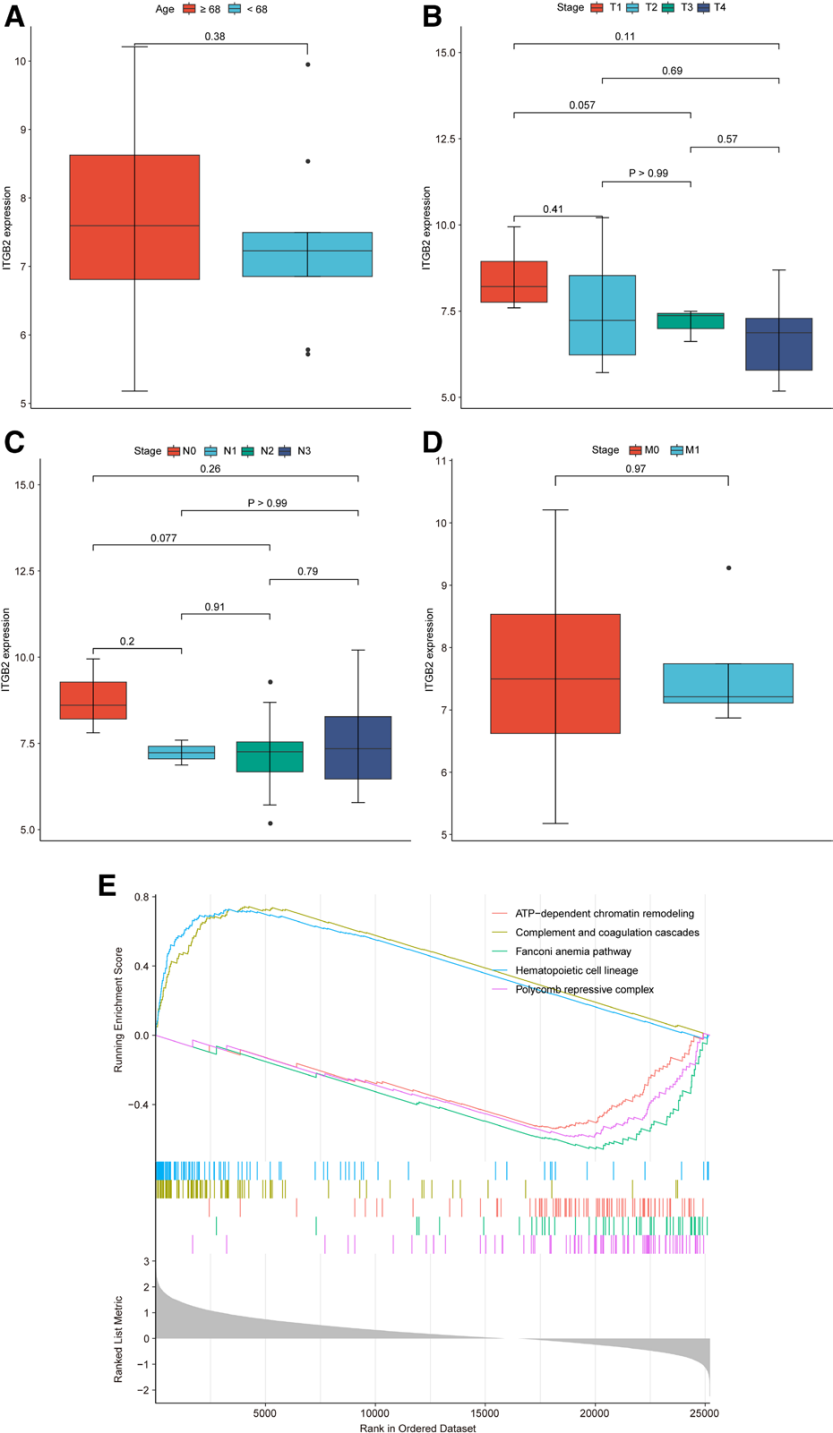
The *ITGB2* expression was not correlated with age and TNM classification. (A) The correlation of *ITGB2* with age. (B–D) The expression of *ITGB2* in TNM classification. (E) The top 5 significantly enriched pathways in *ITGB2*^high^ expression group.

In the GSE30219 dataset, we found that 5 genes (*PTPRC*, *ITGB2*, *CD86*, *CD8A*, and *TLR4*) were significantly correlated with the overall survival of SCLC patients among these 10 genes (Fig. 6A). Among these 5 genes, the low *ITGB2* expression was observed correlated with shorter survival rate of SCLC patients (Fig. 6B). Thus, *ITGB2* was selected as prognostic biomarker of SCLC for subsequent analysis. Meanwhile, in the GSE60052 dataset, SCLC patients with low *ITGB2* expression also had a poor prognosis (Fig. 6C). It has been reported that *ASCL1*, *NEUROD1*, *YAP1*, and *POU2F3* are 4 critical transcription regulators for SCLC. SCLC subtypes can be classified by considering the relative expression of these 4 factors.^[31]^ Thus, we performed a multivariate Cox regression analysis including *ITGB2*, *ASCL1*, *NEUROD1*, *YAP1*, *POU2F3*, age, sex, and stage in the GSE60052 dataset. The results showed that *ITGB2* remained significantly associated with prognosis of SCLC (Fig. 6D, HR = 0.69, *P* = .046). Collectively, *ITGB2* was an independent prognostic factor for survival of SCLC patients. Moreover, in the GSE30219 dataset, *ITGB2* expression was not significantly correlated with age and TNM classification of SCLC patients (Fig. 7A–D). In the GSE60052 dataset, we identified the differentially expressed genes between *ITGB2*^high^ and *ITGB2*^low^ groups to perform the gene-set enrichment analysis. There were 143 signaling pathways were significantly enriched in *ITGB2*^high^ group compared to *ITGB2*^low^ group (Table S4, Supplemental Digital Content, http://links.lww.com/MD/O353), including ATP-dependent chromatin remodeling, complement and coagulation cascades, and Fanconi anemia pathway (Fig. 7E).

### 3.5. ITGB2 involved in the immune cell infiltration and immunotherapy response of SCLC

**Figure 8.**
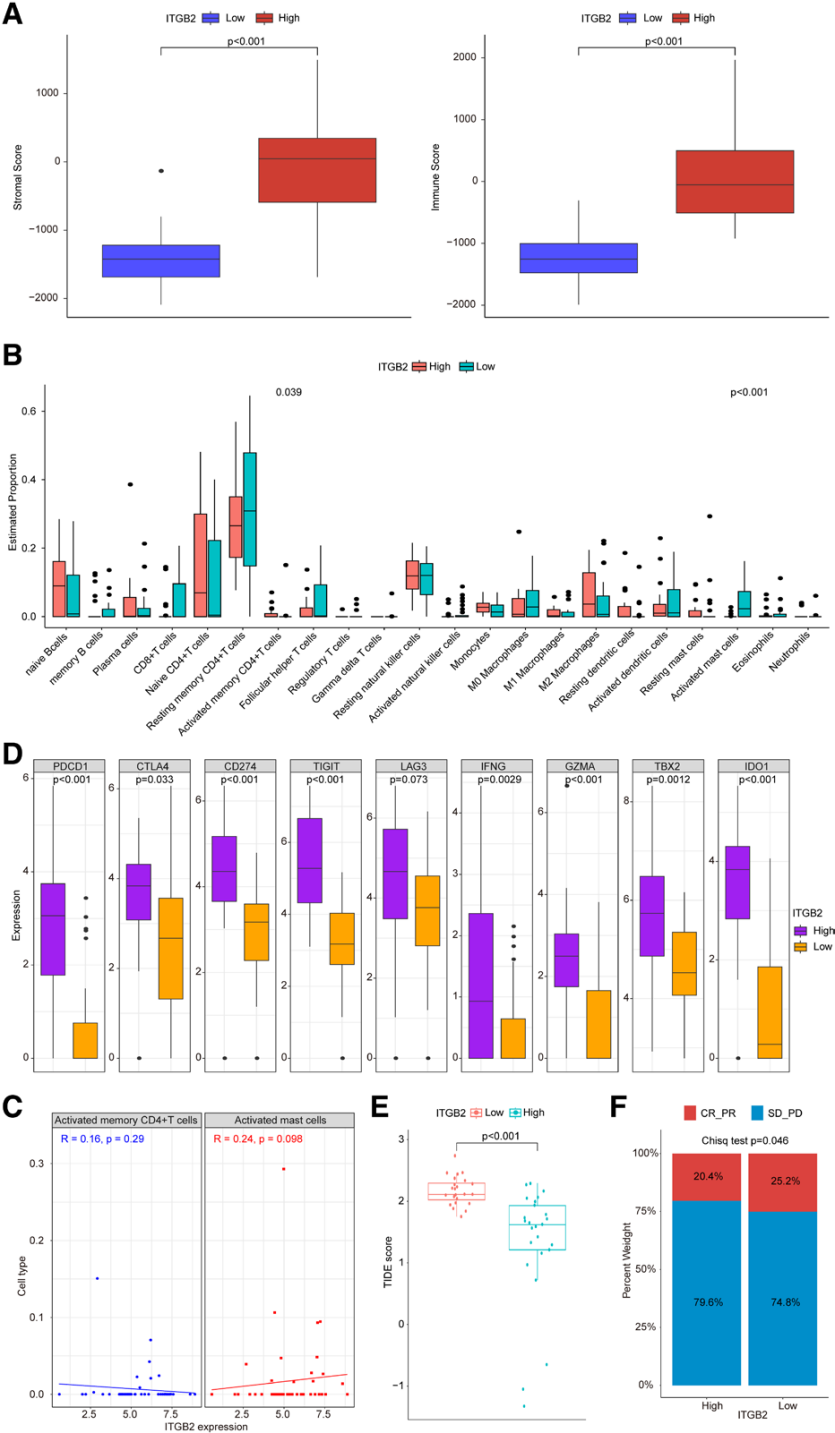
The correlation of *ITGB2* with tumor microenvironment and immunotherapy prediction. (A) The ImmuneScore and StromalScore in *ITGB2*^high^ and *ITGB2*^low^ groups. (B) The infiltration of 22 immune cells in *ITGB2*^high^ and *ITGB2*^low^ groups. (C) The correlation of *ITGB2* with T cells CD4 memory activated and Mast cells activated. (D) The expression of 9 immune checkpoints in *ITGB2*^high^ and *ITGB2*^low^ groups. (E) The correlation of TIDE score in *ITGB2*^high^ and *ITGB2*^low^ groups. (F) The proportion of patients with complete response/partial response (CR/PR) in *ITGB2*^high^ and *ITGB2*^low^ groups. TIDE = tumor immune dysfunction and exclusion.

According to the median expression value of *ITGB2*, SCLC samples were split to *ITGB2*^high^ and *ITGB2*^low^ groups. As shown in Figure 8A, compared to *ITGB2*^low^ group, ImmuneScore and StromalScore were significantly higher in *ITGB2*^high^ group. The infiltration proportion of activated memory CD4 + T cells was significantly increased in *ITGB2*^high^ group and the infiltration proportion of activated mast cells was observably decreased (Fig. 8B). However, *ITGB2* had no significant correlation with the infiltration of activated memory CD4 + T cells and activated mast cells (Fig. 8C).

To explore whether *ITGB2* could affect the immunotherapy response outcome of cancer patients, we first analyzed the expression of immune checkpoints and TIDE score in *ITGB2*^high^ and *ITGB2*^low^ groups. The expression of *PD-1* (*PDCD1*), *CTLA4*, *PDL-1* (*CD274*), *TIGIT*, *IFNG*, *GZMA*, *TBX2*, and *IDO1* were significantly increased in *ITGB2*^high^ group (Fig. 8D, high vs low). The TIDE score was reduced in *ITGB2*^high^ group than that in *ITGB2*^low^ group (Fig. 8E). In addition, we divided urothelial cancer samples in the IMvigor210 cohort into *ITGB2*^high^ and *ITGB2*^low^ groups according to the median *ITGB2* expression. The results showed that the proportion of patients with complete response/partial response in *ITGB2*^high^ group was observably decreased than that in *ITGB2*^low^ group (Fig. 8F). Finally, we analyzed the correlation between *ITGB2* and IC50 of drugs, and found that *ITGB2* exhibited significant negative correlation with IC50 of 121 drugs (Figure S4A, Supplemental Digital Content, http://links.lww.com/MD/O352, Table S5, Supplemental Digital Content, http://links.lww.com/MD/O353, *P* < .05), and had significant positive association with IC50 of 16 drugs (Figure S4B, Supplemental Digital Content, http://links.lww.com/MD/O352, Table S5, Supplemental Digital Content, http://links.lww.com/MD/O353, *P* < .05). These findings indicated that SCLC patients with high *ITGB2* expression had a poor response to immunotherapy, and *ITGB2* might be a potential target gene of drug in cancer.

## 4. Discussion

In this study, 10 hub genes (*PTPRC*, *RPS27A*, *UBA52*, *CD8A*, *ITGB2*, *GNB2L1*, *TYROBP*, *CD86*, *TLR4*, and *FCGR3A*) associated with CD8 + T cells were identified by WGCNA and PPI network analyses. Among which, *ITGB2*, *GNB2L1*, and *FCGR3A* were down-regulated in SCLC samples, and *ITGB2* was positively correlated with CD8 + T cells in SCLC. Furthermore, the down-regulation of *ITGB2* was found to be associated with poorer prognosis of SCLC patients.

*ITGB2*, a member of the integrin family, has been documented to exhibit predominant expression on immune cells. In oral squamous cell carcinoma, *ITGB2* was highly expressed in cancer associated fibroblasts (CAFs) compared to normal fibroblasts, and the up-regulation of *ITGB2* in CAFs was associated with more Ki67 + tumor cells, which suggested that *ITGB2* could promote the proliferation of oral squamous cell carcinoma.^[32]^ We found that *ITGB2* expression was positively correlated with CD8 + T cells in SCLC. CD8 + T cells was one of the common prevalent T cells subtypes^[33]^ and the triple-therapy of niraparib, RT, and anti-PD-1 could improve the antitumor immunity by enhancing cytotoxic CD8 + T cells in the TME.^[20]^ In NSCLC, the patients with high level of CD8 + T cells exhibited better prognosis.^[34]^ Thus, *ITGB2* might be a CD8 + T cells infiltration marker in lung cancer patients. In addition, previous researches have reported that *ITGB2* expression participated in the progression, prognosis and tumor stage. Liu et al found that the *ITGB2-AS1* could up-regulate *ITGB2* to enhance the migration and invasion of breast cancer cell.^[35]^ In triple negative breast cancer, *ITGB2* overexpression was correlated with the tumor stage and metastasis.^[36]^ In the present study, the expression of *ITGB2* was not observably correlated with age and TNM classification in SCLC. In chronic lymphocytic leukemia, high expression of *ITGB2* promoted the growth of cancer cells.^[37]^ Wei et al indicated that the level of ITGB2 protein was increased in acute myeloid leukemia samples compared to normal samples, and the acute myeloid leukemia patients with high expression of *ITGB2* displayed worse outcome.^[38]^ In glioma patients with higher malignancy, *ITGB2* expression was elevated and showed worse prognosis and the patients with high level of *ITGB2* mRNA exhibited better responses to immunotherapy.^[39]^ Zu et al revealed that NSCLC patients with *ITGB2* down-regulation exhibited mediocre overall survival rate,^[40]^ which was consistent with our results that the down-regulation of *ITGB2* was associated with inferior prognosis of SCLC patients. Moreover, the overexpression of *ITGB2* could inhibit the migration and proliferation of NSCLC cells.^[40]^ Accordingly, *ITGB2* might act as a tumor-promoting gene or tumor-suppressor gene in different cancers.

The ImmuneScore and StromalScore were significantly high in *ITGB2*^high^ group. In tumor immune microenvironment, the higher ImmuneScore and StromalScore were associated with the larger number of stromal and immune components.^[41]^ However, *ITGB2* expression had not significant correlation with the infiltration of activated memory CD4 + T cells and activated mast cells. Moreover, the expression of immune checkpoints (*PD-1* (*PDCD1*), *CTLA4*, *PDL-1* (*CD274*), *TIGIT*, *IFNG*, *GZMA*, *TBX2*, *IDO1*) was significantly increased in *ITGB2*^high^ group. Immune checkpoints, as negative regulators in immune system, they participated in the prevented autoimmunity and protected tissues from immune damage.^[42]^ The inhibition of immune checkpoints could enhance the antitumor immunity in same cancers, such as NSCLC^[43]^ and hepatic cancer.^[44]^ In addition, based on the profiles of pretherapeutic cancer, the TIDE score could predict the response to treatment with immune checkpoint blockade.^[45]^ Our results showed that SCLC patients with high *ITGB2* expression had lower TIDE score. In urothelial cancer, we discovered that the proportion of patients with complete response/partial response in *ITGB2*^high^ group was observably decreased than that in *ITGB2*^low^ group. These findings indicated that patients with high *ITGB2* expression were able to poor response to immunotherapy.

## 5. Conclusion

In summary, the *ITGB2* was firstly demonstrated to associate with CD8 + T cells in SCLC, and the patients with low *ITGB2* expression exhibited worse prognosis. In addition, *ITGB2* might correlated with response to immunotherapy in cancer. Our findings indicated that *ITGB2* might serves as a prognostic marker of SCLC patients.

## Author contributions

**Conceptualization:** Wen Tian.

**Data curation:** Wen Tian, Jinhui Zhao.

**Formal analysis:** Nana Wang, Wen Tian, Jinhui Zhao.

**Investigation:** Nana Wang.

**Methodology:** Wen Tian.

**Resources:** Nana Wang.

**Software:** Nana Wang.

**Validation:** Nana Wang, Wen Tian, Wenzhong Wang.

**Visualization:** Nana Wang, Wen Tian, Wenzhong Wang.

**Writing – original draft:** Wen Tian, Jinhui Zhao, Wenzhong Wang, Fu Mi.

**Writing – review & editing:** Fu Mi.

## Supplementary Material


