# Supplementary material for: Significance of CD8+T cells related gene ITGB2 in prognosis and tumor microenvironment of small cell lung cancer
Source: Medicine (Baltimore). 2025 Feb 14;104(7):e41461. doi: 10.1097/MD.0000000000041461 (PMC11835109; doi:10.1097/MD.0000000000041461)

Fig. S1 The PPI network results of 609 genes were visualized by Cytoscape software

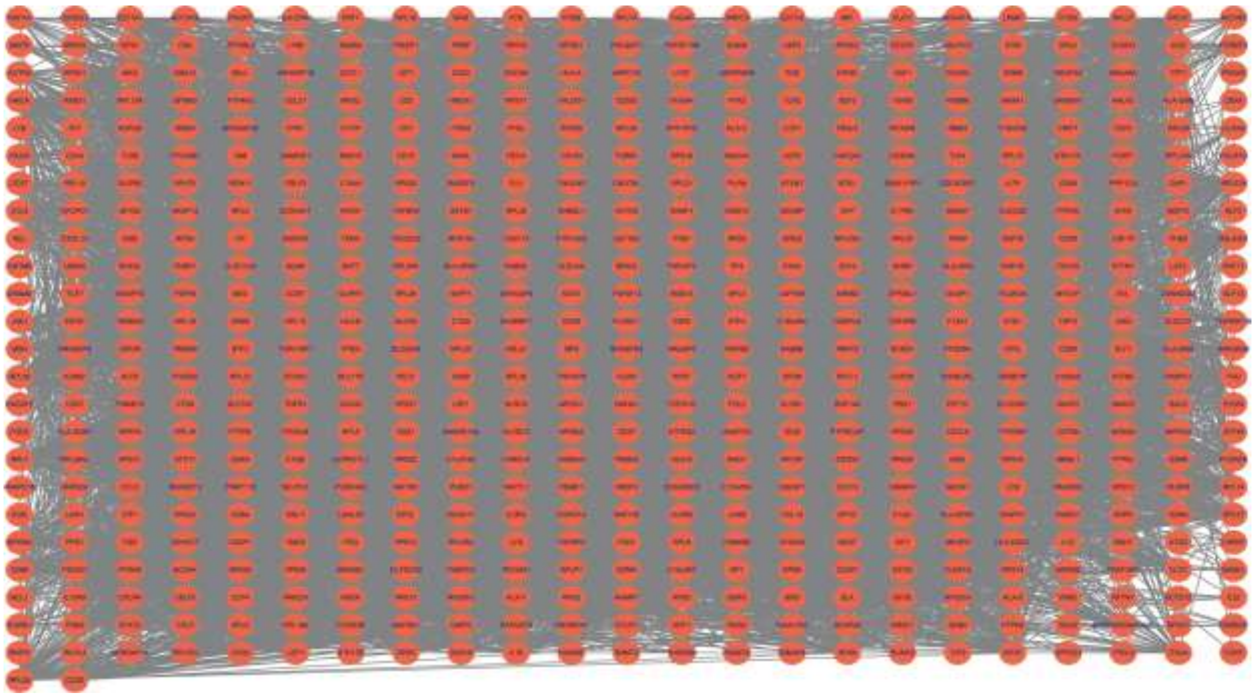

Fig. S2 Univariate cox regression analysis and transcription factor regulatory network. A. The correlation between CD8+T cells and prognosis of SCLC via univariate cox regression analysis in three database. B. The result of transcription factor regulatory network displayed by Cytoscape.

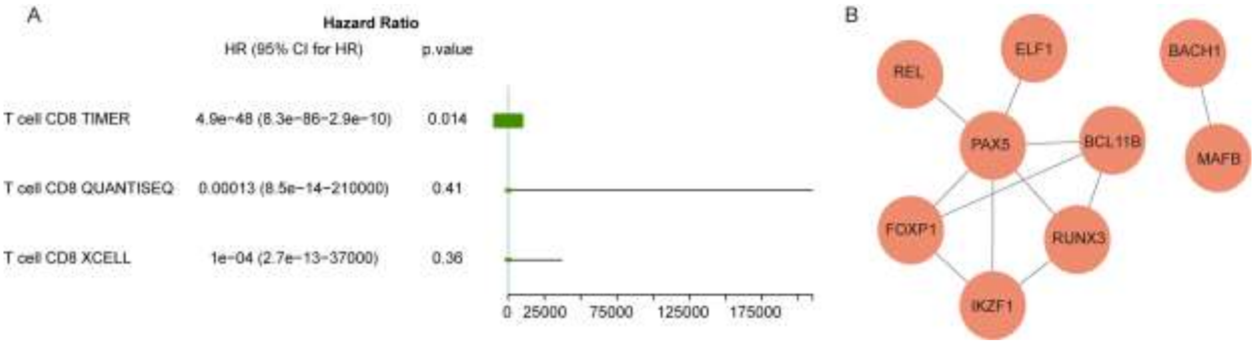

Fig. S3 The correlation between *GNB2L1* (A), *RPS27A* (B), *TLR4* (C), *UBA52* (D) and tumor-infiltrating lymphocytes in pan-carcinoma

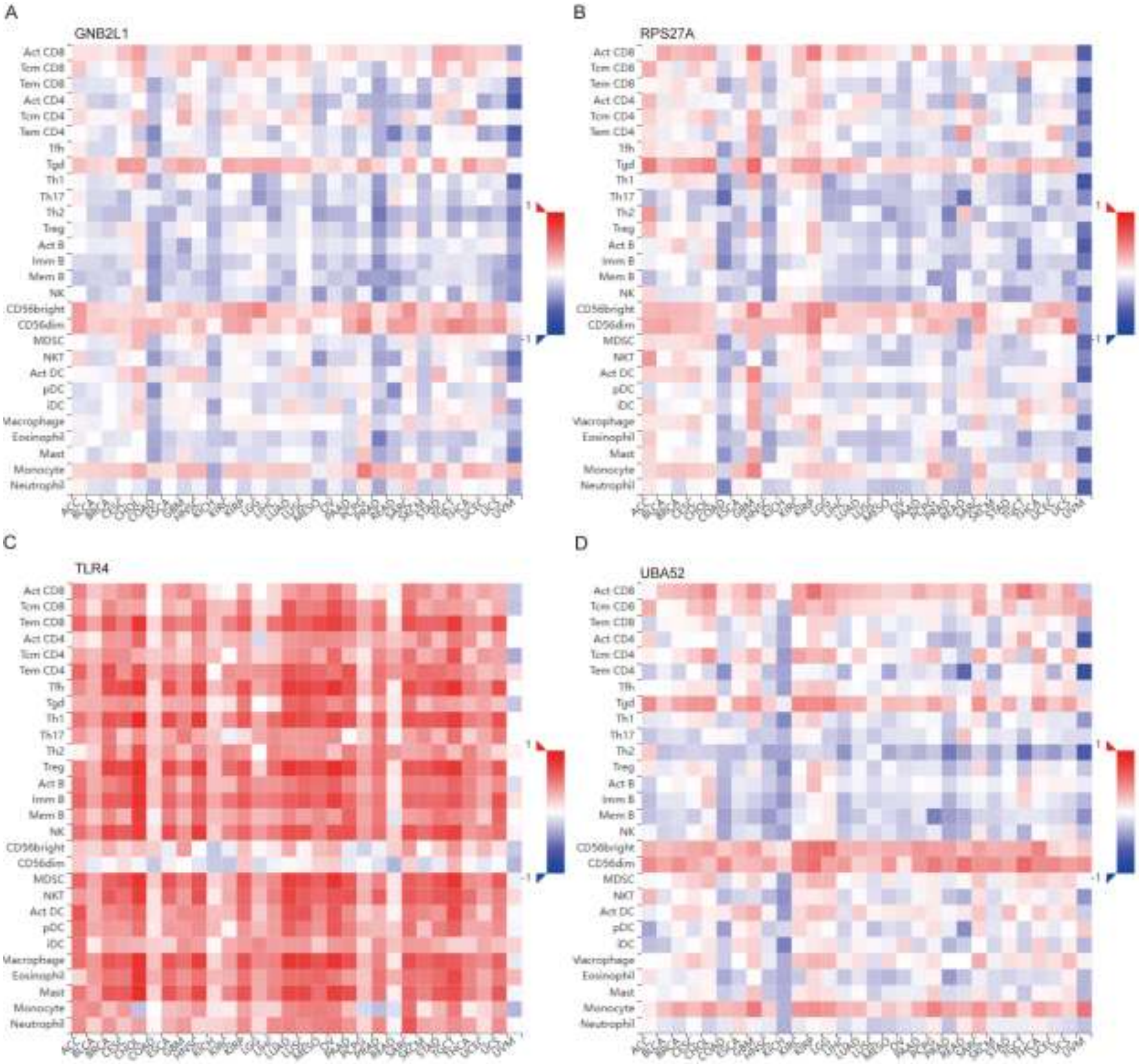

Fig. S4 Drugs negatively (A) and positively (B) associated with *ITGB2*

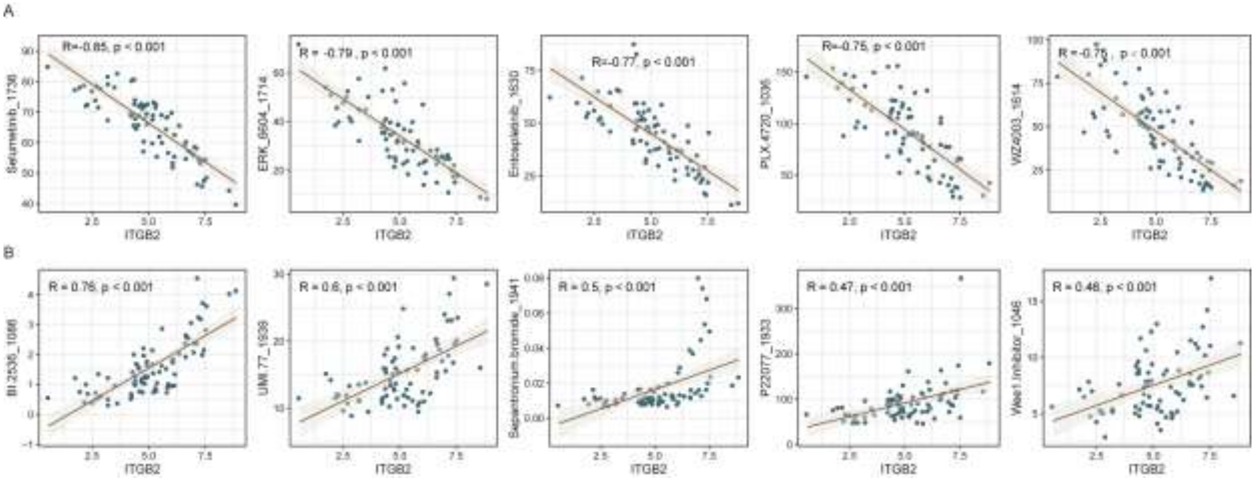

Supplement: Supplementary file 1 [file medi-104-e41461-s001.pdf]
